# Widening Socioeconomic, Racial, and Geographic Disparities in HIV/AIDS Mortality in the United States, 1987–2011

**DOI:** 10.1155/2013/657961

**Published:** 2013-05-07

**Authors:** Gopal K. Singh, Romuladus E. Azuine, Mohammad Siahpush

**Affiliations:** ^1^US Department of Health and Human Services, Health Resources and Services Administration, Maternal and Child Health Bureau, 5600 Fishers Lane, Room 18-41, Rockville, MD 20857, USA; ^2^Department of Health Promotion, Social and Behavioral Health, University of Nebraska Medical Center, Omaha, NE 68198-4365, USA

## Abstract

This study examined the extent to which socioeconomic and racial and geographic disparities in HIV/AIDS mortality in the United States changed between 1987 and 2011. Census-based deprivation indices were linked to county-level mortality data from 1987 to 2009. Log-linear, least-squares, and Poisson regression were used to model mortality trends and differentials. HIV/AIDS mortality rose between 1987 and 1995 and then declined markedly for all groups between 1996 and 2011. Despite the steep mortality decline, socioeconomic gradients and racial and geographic disparities in HIV/AIDS mortality increased substantially during the study period. Compared to whites, blacks had 3 times higher HIV/AIDS mortality in 1987 and 8 times higher mortality in 2011. In 1987, those in the most-deprived group had 1.9 times higher HIV/AIDS mortality than those in the most-affluent group; the corresponding relative risks increased to 2.9 in 1998 and 3.6 in 2009. Socioeconomic gradients existed across all race-sex groups, with mortality risk being 8–16 times higher among blacks than whites within each deprivation group. Dramatic reductions in HIV/AIDS mortality represent a major public health success. However, slower mortality declines among more deprived groups and blacks contributed to the widening gap. Mortality disparities reflect inequalities in incidence, access to antiretroviral therapy, and patient survival.

## 1. Introduction

Social inequalities in health, disease, and mortality have long represented an important area of public health research in the United States [1–4]. Previous research has shown the dynamic nature of health inequalities in the USA, with social inequalities in health and many disease outcomes either persisting or widening over time against a backdrop of consistent improvements in life expectancy and mortality among Americans [1, 3–7]. Reduction of health inequalities, including those between socioeconomic and racial/ethnic groups, has been an important policy goal for the United States since 1990, as specified in its national health initiative, *Healthy People* [3, 6, 8–10].

Trends and contemporary patterns in HIV/AIDS mortality are routinely analyzed by age, sex, race/ethnicity, and state of residence in the United States [1, 11–13]. A few US studies have also examined differentials in HIV/AIDS mortality according to socioeconomic status (SES) or area-based deprivation level [14–16]. However, studies of temporal socioeconomic and geographic disparities in HIV/AIDS mortality are quite rare in the USA [14–16]. 

According to the Centers for Disease Control and Prevention, HIV incidence and mortality rates are substantially higher in the Southern and Northeastern United States and among blacks and Hispanics than whites [11, 17–19]. In 2009, the rate of new HIV infections was 8 times higher for blacks and 3 times higher for Hispanics than whites and Asians; the HIV/AIDS mortality rate was 9 times higher for blacks than for whites [11, 17]. Racial disparities were more marked among women, with black women having a 15 times higher rate of new HIV infections than white women and over 3 times higher rate than Hispanic women [17].

To our knowledge, comprehensive efforts have not yet been undertaken to examine socioeconomic patterns in HIV/AIDS mortality across major race-sex groups in a temporal fashion, using annual mortality data from 1987 through 2011 and covering the entire age range including children and adults. It is important to estimate and monitor the magnitude of HIV/AIDS mortality disparities across racial and socioeconomic groups and geographic areas for the purposes of setting up HIV education and prevention programs, resource allocation, and target setting. While temporal analysis allows us to track progress toward reducing health inequalities among those at higher risks of morbidity and mortality, it also provides important insights into the role of health policy and medical interventions, such as the introduction of highly active antiretroviral therapy (HAART) in lowering HIV/AIDS mortality since 1996, differential access to health services, and changing socioeconomic conditions [3–5, 14]. 

The aim of our study is to examine changes in the extent of socioeconomic, racial, and geographic disparities in HIV/AIDS mortality in the USA between 1987 and 2011. We use census-based deprivation indices linked to national vital statistics mortality data to examine the extent to which area-based socioeconomic differences in HIV/AIDS mortality have changed since the USA began recording HIV/AIDS mortality statistics for the entire nation in 1987. We examine annual trends in black/white disparities in HV/AIDS mortality and analyze whether socioeconomic gradients in HIV/AIDS mortality vary by race and sex.

## 2. Methods

To analyze temporal inequalities in HIV/AIDS mortality, we used the national vital statistics mortality database [1, 10–12, 20]. Since the vital-statistics-based national mortality database lacks reliable SES data, socioeconomic patterns in HIV/AIDS mortality were derived by linking the 1990 and 2000 census-based county-level deprivation indices to the age-sex-race-county-specific mortality statistics from 1987 through 2009 [3–7, 21]. 

**Figure 1:**
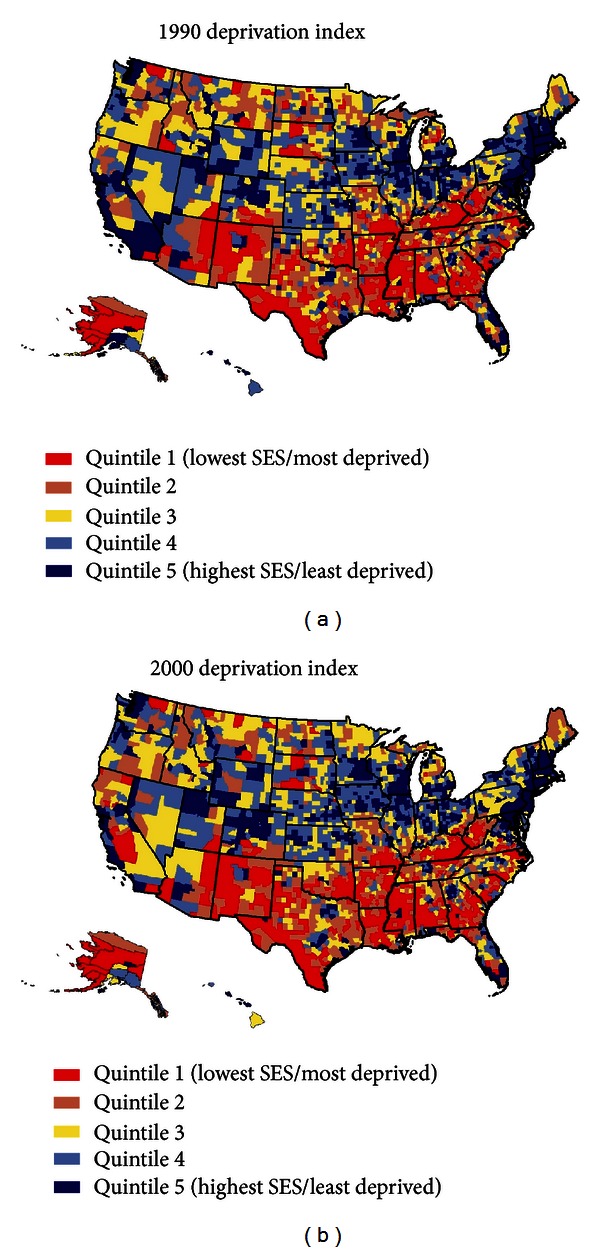
Area (county) socioeconomic deprivation indices, United States.

We used two previously published factor-based deprivation indices from the 1990 and 2000 decennial US censuses [4–6, 21]. The 1990 deprivation index consisted of 17 census-based socioeconomic indicators, which may be viewed as broadly representing educational opportunities, labor force skills, and economic and housing conditions in a given county [4–6]. Selected indicators of education, occupation, wealth, income distribution, unemployment rate, poverty rate, and housing quality were used to construct the 1990 index [4–6]. The factor loadings (correlations of indicators with the index) for the 1990 index ranged from 0.92 for 150% of the poverty rate to 0.45 for household plumbing [4–6]. The 2000 deprivation index consisted of 22 socioeconomic indicators, including five additional measures of income distribution, wealth, and housing quality [21]. The factor loadings for the 2000 index varied from 0.92 for 150% of the poverty rate to 0.39 for household plumbing [21]. The common indicators in the 1990 and 2000 deprivation indices generally had similar factor loadings or relative weights [4–6, 21]. The geographical distribution of deprivation was similar in 1990 and 2000; the correlation between the 1990 and 2000 deprivation indices was 0.97 (Figure 1). Substantive and methodological details of the US deprivation indices are provided elsewhere [3–6, 21].

In order to compute mortality rates by deprivation level, we used the weighted population quintile distribution of the deprivation index that classified all 3,141 US counties into 5 groups of approximately equal population size [4–6, 21]. The groups thus created ranged from being the most-deprived (first quintile) to the least-disadvantaged (fifth quintile) population groups [4–6, 21]. A majority of the deprived counties were concentrated in the Southeastern region, whereas many of the affluent counties were located in the Northeastern and Western regions of the United States (Figure 1). Each of the 3,141 counties in the mortality database was assigned one of the 5 deprivation quintiles. The 1990 index was used to compute deprivation-specific mortality rates from 1987 to 1998, whereas the 2000 index was used to compute mortality rates by deprivation level from 1999 to 2009. To simplify analysis and data presentation, we combined the third, fourth, and fifth quintiles of the deprivation index since mortality rates did not vary greatly among the middle deprivation categories.

While HIV/AIDS mortality rates were computed annually for the total population and by race and sex between 1987 and 2011, mortality rates by county-deprivation level were computed annually between 1987 and 1998 and for the following time periods: 1999–2001, 2002–2004, and 2005–2009. County-specific mortality statistics were available in an annual fashion from 1987 to 1998 and in three-year and five-year periods from 1999 onwards. Age-sex-race-county-specific HIV/AIDS deaths for the above time periods were obtained from the national mortality database [1, 11–13, 20] whereas age-sex-race-county-specific populations for the same time periods were developed by the US Census Bureau and served as denominators for computing mortality rates [11–13, 22–24]. Our analysis included all 479,833 HIV/AIDS deaths that occurred in the USA between 1987 and 2011. Of these, 267,494 (or 55.7%) deaths occurred among whites and 207,285 (or 43.2%) deaths among blacks.

 Mortality rates for each county, area-socioeconomic group, and race-sex group were age-adjusted by the direct method using the age composition of the 2000 US population as the standard and age-specific mortality rates for 19 age groups: <1, 1–4, 5–9, 10–14,…  ,  80–84, 85+ years [4, 7, 11]. Log-linear regression models were used to estimate annual rates of decline in HIV/AIDS mortality for race and deprivation groups [3–7]. Specifically, the logarithm of mortality rates was modeled as a linear function of time (calendar year), which yielded annual exponential rates of change in mortality rates [3–7]. Race and deprivation disparities in mortality were described by rate ratios (relative risks) and rate differences (absolute inequalities), which were tested for statistical significance at the 0.05 level. Additionally, spatial inequalities in HIV/AIDS mortality were analyzed in 1992–1995 and 2003–2007. County-level variations in age-adjusted HIV/AIDS mortality rates in 1992–1995 and 2003–2007 were modeled by weighted least-squares regression as a function of the continuous deprivation index, urbanization level, health professional shortage area for primary medical care services, racial composition, and health uninsurance rate [25, 26]. Poisson regression models, as estimated by the SAS GENMOD procedure, were used to derive relative risks of HIV/AIDS mortality using the same covariates as in the least-squares models [25].

## 3. Results

**Figure 2:**
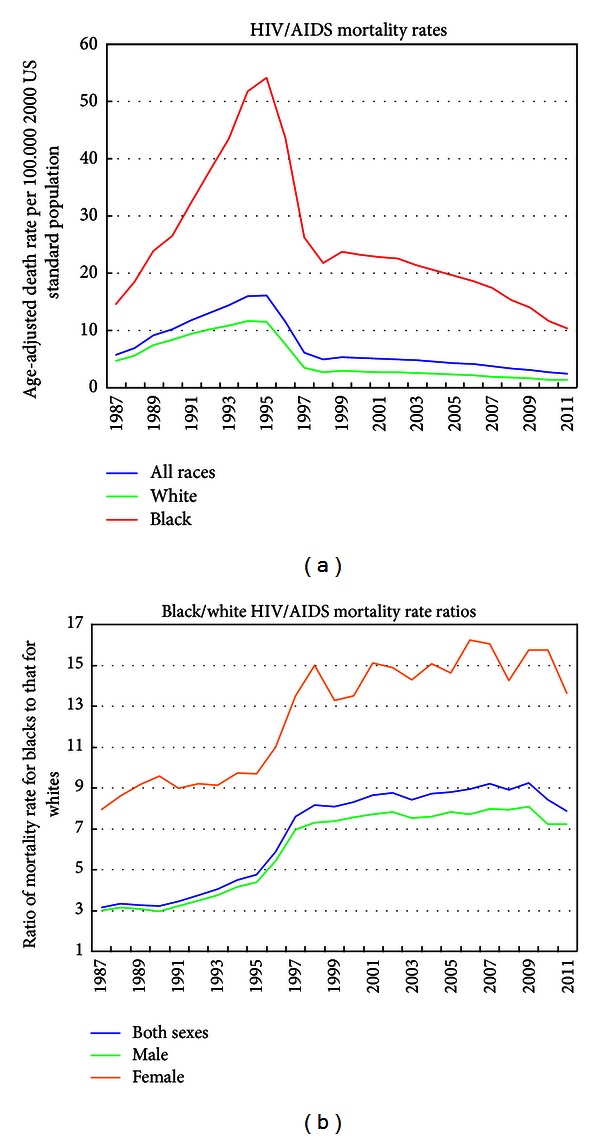
Trends in age-adjusted HIV/AIDS mortality rates and rate ratios by race, United States, 1987–2011.

Figure 2 presents the annual HIV/AIDS mortality trends from 1987 to 2011 for the total US population and for blacks and whites. HIV/AIDS mortality rose steeply from 1987 through 1995, the peak of the disease epidemic, and then began a steep decline, with the mortality rate dropping from 16.1 deaths per 100,000 population in 1995 to 2.4 in 2011. While absolute black/white differences in HIV/AIDS mortality, as measured by rate differences, narrowed between 1995 and 2011, racial disparities in HIV/AIDS mortality, as measured by rate ratios, widened consistently between 1987 and 2011 (Figure 2). Compared to whites, blacks had 3.1 times higher HIV/AIDS mortality in 1987, 4.7 times higher mortality in 1995, and 7.9 times higher mortality in 2011. Racial disparities were particularly marked among women, with black women having 7.9 times higher HIV/AIDS mortality in 1987, 9.7 times higher mortality in 1995, and 15.7 times higher mortality in 2010 than white women (Figure 2). Relative black/white disparities in HIV/AIDS mortality increased over time because HIV/AIDS mortality among blacks rose faster during 1987–1995 and declined more slowly during 1995–2011 than whites. During 1987–1995, HIV/AIDS mortality rates increased by 12.0% per year for whites and by 18.0% per year for blacks. During 1995–2011, HIV/AIDS mortality among whites and blacks decreased by 9.2% and 7.2% annually, respectively. Among all race-sex groups, black women experienced the slowest decline in their HIV/AIDS mortality between 1995 and 2011.

**Figure 3:**
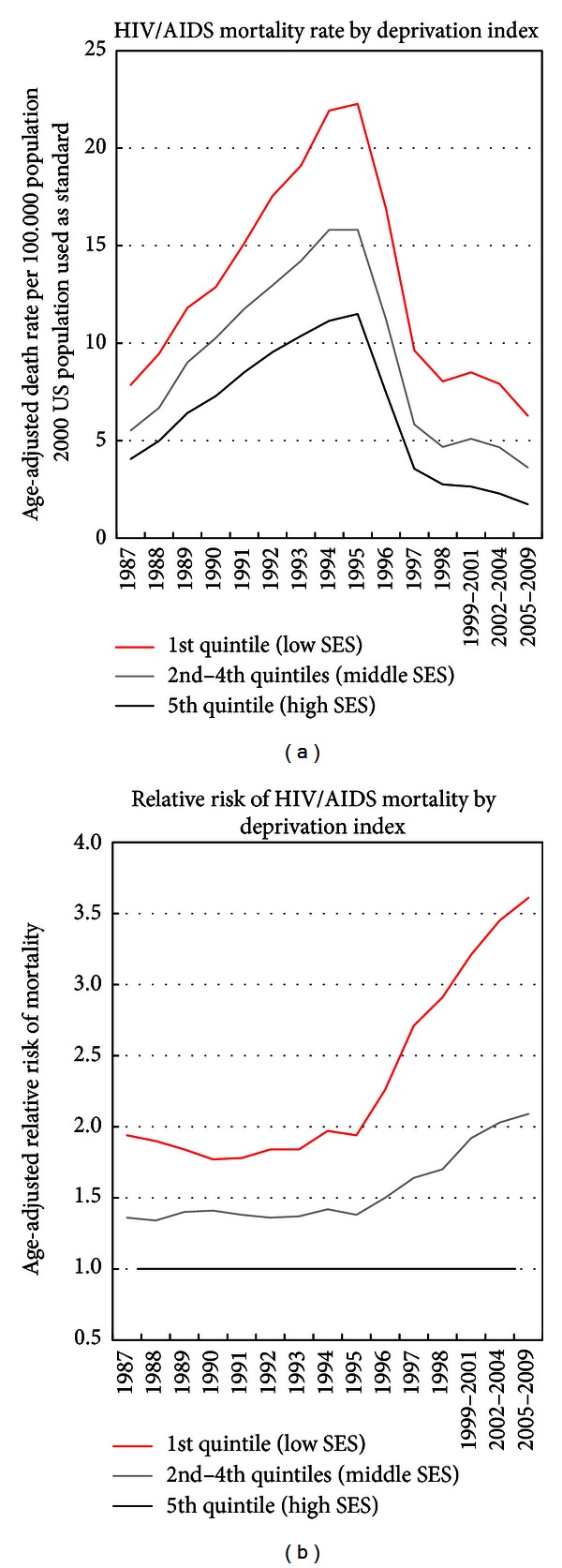
Trends in HIV/AIDS mortality by area socioeconomic deprivation index, both sexes combined, United States 1987–2009.

Although HIV/AIDS mortality rates declined over time for all deprivation groups, more deprived groups had higher mortality than less deprived groups in each time period, and the SES gradients (rate ratios) increased over time, particularly since 1996 (Figure 3 and Table 1). In 1987, those in the most-deprived group had 1.9 times higher HIV/AIDS mortality than those in the most-affluent group; the corresponding relative mortality risks increased to 2.9 in 1998 and 3.6 in 2005–2009. During 1995–2009, HIV/AIDS mortality in the three most-to-least deprived groups declined at average annual rates of 8.2%, 9.4%, and 12.4%, respectively. HIV/AIDS mortality in the three deprivations groups had risen by 14% annually during 1987–1995.

**Figure 4:**
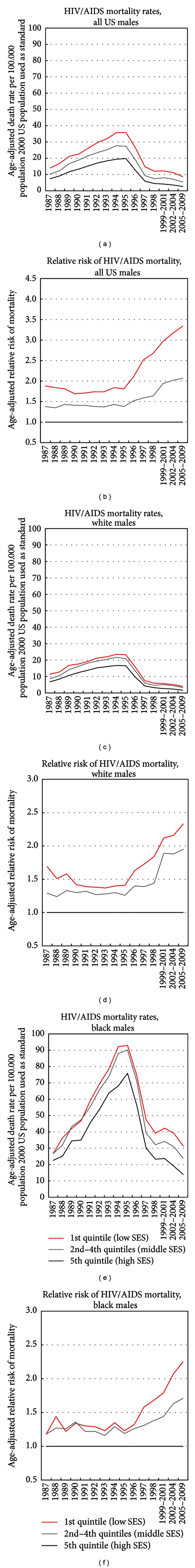
Trends in race-specific HIV/AIDS mortality by area socioeconomic deprivation, US males, 1987–2009.

**Figure 5:**
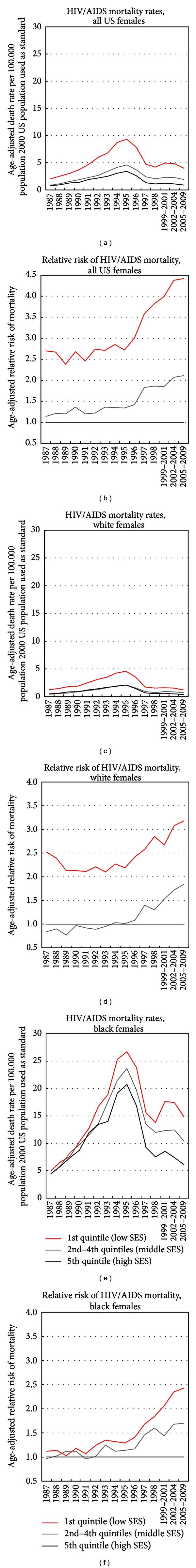
Trends in race-specific HIV/AIDS mortality by area socioeconomic deprivation, US females, 1987–2009.

HIV/AIDS mortality rates increased with increasing deprivation levels for males and females and for whites and blacks, with the SES gradients in mortality increasing consistently for all race-sex groups between 1996 and 2009 (Figures 4 and 5). For example, compared to their affluent counterparts, black men in the most-deprived group had 1.18 times higher mortality in 1987, 1.7 times higher mortality in 1998, and 2.3 times higher mortality in 2005–2009. For all race-sex groups, the rate of decline in HIV/AIDS mortality during 1995–2009 was significantly faster in the most-affluent group than in the more deprived groups. During 1995–2009, HIV/AIDS mortality among black men and women in the most-deprived group declined, respectively, by 7.2% and 3.2% annually, whereas HIV/AIDS mortality among white men and women in the most-affluent group declined by 14.9% and 11.0% per year, respectively.

**Figure 6:**
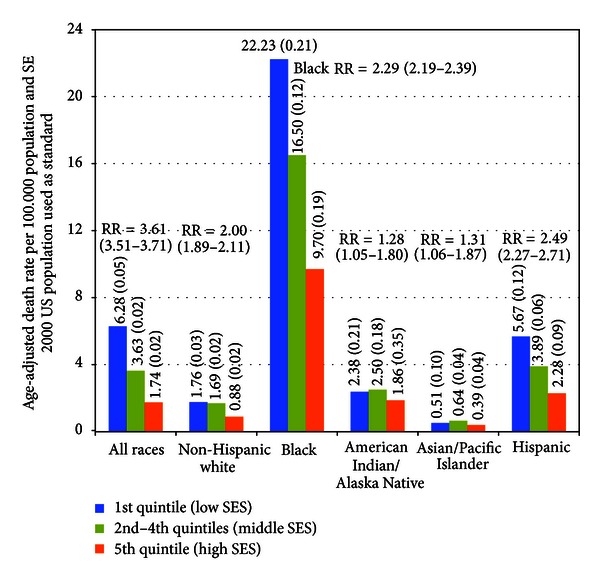
HIV/AIDS mortality by race/ethnicity and socioeconomic deprivation level, United States, 2005–2009. RR is ratio of mortality rate for the 1st quintile (low SES) to that for the 5th quintile (high SES). The standard normal *Z* test can be used to evaluate the statistical significance of the difference in mortality rates between any two groups at one point in time or to test for change in mortality rates between two time points for a specific group. If the absolute value of the *Z*-statistic = (R1 − R2)/SQRT[(SE  (R1))^2^ + (SE  (R2))^2^] ≥ 1.96, then the difference is statistically significant at the 0.05 level. If the test statistic ≥2.58, then the difference in the rates is significant at the 0.01 level, where R1 is mortality rate for the first demographic group, R2 is mortality rate for the second demographic group, and SE (R1) and SE (R2) are the standard errors associated with R1 and R2, respectively. For example, during 2005–2009, the HIV/AIDS mortality rate for Hispanics in the most-deprived socioeconomic group (5.67) was 3.2 times higher than the corresponding rate for non-Hispanic whites (1.76), and this difference was statistically significant at the 0.01 level (computed *Z*-value = 31.61). The 95% confidence interval for a specific mortality rate can be constructed by using the formula = rate ± 1.96 * standard error.

Substantial socioeconomic differences in HIV/AIDS mortality are also evident for detailed racial/ethnic groups in the USA (Figure 6). In 2005–2009, mortality rates varied from a low of 0.4 deaths per 100,000 population for Asians/Pacific Islanders in the least-deprived group to a high of 22.2 for blacks in the most-deprived group. Socioeconomic gradients were steepest for Hispanics, blacks, and non-Hispanic whites. Non-Hispanic whites, blacks, Hispanics, Asian/Pacific Islanders, and American Indians/Alaska Natives had, respectively, 2.0, 2.3, 2.5, 1.3, and 1.3 times higher HIV/AIDS mortality in the most-deprived group than in the least-deprived group (Figure 6).

**Figure 7:**
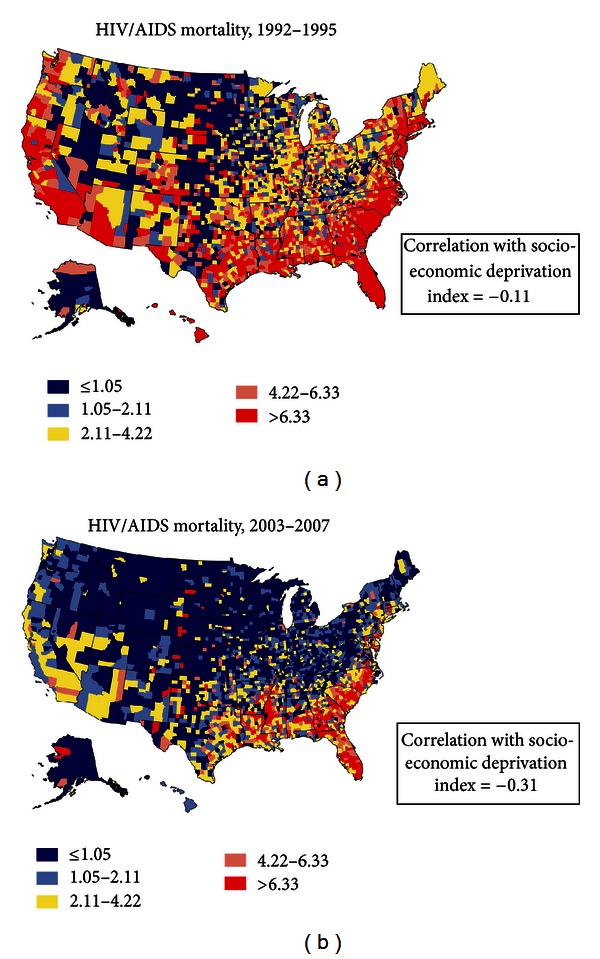
Age-adjusted HIV/AIDS mortality rates per 100,000 population, 1992–2007 (2000 US population used as standard; 3,141 counties).

Geographic disparities in HIV/AIDS mortality were measured by the coefficient of variation (CV), which increased between 1992–1995 and 2003–2007 for the total population (from 840.8 to 853.1), all blacks (from 181.4 to 208.9), black males (from 117.8 to 127.5), and black females (from 178.4 to 190.4). A comparison of the county maps in 1992–1995 and 2003–2007 shows substantial reductions in mortality for most counties and geographic regions, with higher HIV/AIDS mortality being concentrated in the Southeastern region ofUSA during 2003–2007 (Figure 7). Geographical distributions in SES and HIV/AIDS mortality rates were more closely related in 2003–2007 than in 1992–1995, with the weighted correlations being −0.31 in 2003–2007 and −0.11 in 1992–1995 (Figure 7 and Table 2); higher deprivation levels and HIV/AIDS mortality were both seen in the Southeastern region (Figures 1 and 7). 

Socioeconomic deprivation, urbanization levels, health professional shortage area, racial composition, and health insurance coverage rates were all independently and significantly related to higher rates and risks of HIV/AIDS mortality (Tables 2 and 3). These covariates jointly accounted for 53.5% of the variance in HIV/AIDS mortality rates in 2003–2007 (Table 2). During 2003–2007, each 20-point increase in the deprivation level was associated with a 0.80-point increase in the HIV/AIDS mortality rate, whereas a 5-percentage-point increase in the health insurance rate was associated with a 0.50-point decrease in the mortality rate (Table 2, multivariate models). Racial composition (as measured by the percent of black population) had the greatest impact on HIV/AIDS mortality, followed by urbanization level, socioeconomic deprivation, and health insurance (Table 2). According to the multivariate Poisson models, those in the most-deprived group in 2003–2007 had a 2.3 times higher risk of HIV/AIDS mortality than those in the most-affluent group; those in large metropolitan areas had 3.9 times higher mortality risk than their rural counterparts; and those in the underserved areas had 28% higher mortality risk than those in nonshortage areas (Table 3). Each 5-percentage-point increase in the uninsured population and a 10-percentage-point increase in the black population were associated with 14% and 38% higher HIV/AIDS mortality, respectively (Table 3). Generally similar effects of covariates on HIV/AIDS mortality were observed for black males and females in 2003–2007 (Tables 2 and 3). In 1992–1995, although higher deprivation levels were generally associated with higher HIV/AIDS mortality, controlling for the covariates attenuated the mortality impact of deprivation. The effects of urbanization, health insurance, health professional shortage areas, and racial composition remained significant (Tables 2 and 3). The impact of deprivation on HIV/AIDS mortality was weaker while the impact of urbanization was stronger in 1992–1995 than in 2003–2007 (Tables 2 and 3). 

## 4. Discussion

Although HIV/AIDS mortality rates have declined sharply for all sociodemographic groups in the United States since 1996 [1, 11], our study shows substantial and increasing socioeconomic, racial, and geographic disparities in HIV/AIDS mortality over time, as relative inequalities in mortality widened consistently between 1987 and 2011. Between 1995 and 2009, whites and those in the most-affluent group experienced faster declines in mortality than blacks and those in the most-deprived group, which contributed to the widening racial and socioeconomic gap in HIV/AIDS mortality. However, absolute inequalities in HIV/AIDS mortality, as measured by mortality rate differences, between blacks and whites and across deprivation groups have narrowed over time, particularly since 1996. The narrowing of the absolute inequalities in mortality between social groups and dramatic reductions in HIV/AIDS mortality among all race and socioeconomic groups represent a major public health success. 

The impact of deprivation on HIV/AIDS mortality was substantial, particularly among US women. Deprivation was related to higher HIV/AIDS mortality among all race-sex groups, with marked racial disparities continuing to exist regardless of deprivation level. When both race and deprivation are taken into account, the inequalities are found to be even more marked, with blacks experiencing 8–16 times higher mortality risk than whites within each deprivation group. Remarkably, the current HIV/AIDS mortality experience of black men is similar to the mortality experience of whites in the mid-1990s. Although HIV/AIDS mortality rates for black women declined by 72% between 1995 and 2011, their rate of 6.8 per 100,000 population in 2011 was still three times higher than the mortality risk that white women experienced at the peak of the HIV/AIDS epidemic in 1995 and prior to the introduction of HAART.

The temporal patterns in HIV/AIDS mortality are consistent with those observed for all-cause mortality and mortality from several major causes of death in the USA, where racial and socioeconomic disparities (in terms of relative inequalities) have increased over time even as overall mortality rates have declined markedly [3–7, 27]. Existence of such marked and growing socioeconomic and racial disparities in HIV/AIDS mortality in the USA runs counter to the goals of the national health initiative that calls for further reductions or elimination of health inequalities by 2020 [8]. 

Increasing socioeconomic and racial disparities in HIV/AIDS mortality shown here are consistent with those observed previously for the United States and Europe [14–16, 28–30]. The substantial reductions in HIV/AIDS mortality in both the USA and Europe have been attributed to the introduction of HAART in 1996, which, according to the previous studies, have led to the widening of socioeconomic differences in HIV/AIDS mortality over time in relative terms [14–16, 28–30]. Studies have interpreted these persistent or growing social inequalities in HIV/AIDS mortality in the post-HAART periods as partly indicative of lower access or adherence to HAART treatment among lower SES groups [14, 28–30].

Disparities in HIV/AIDS mortality shown here most likely reflect inequalities in HIV incidence, access to antiretroviral therapy, and patient survival [21]. Consistent with the patterns in HIV/AIDS mortality reported here, marked socioeconomic disparities have been found in AIDS incidence in Massachusetts, with those in the most-deprived neighborhood group having a 7 times higher incidence rate than those in the least-deprived group [31]. In this study, consistent SES gradients in incidence were found for all racial/ethnic groups, with blacks and Hispanics having higher incidence rates than whites in each deprivation group [31]. Ethnic minority groups and people with low SES have been found to have lower access to health services, including treatment with HAART [32]. Moreover, socioeconomic differences in patient survival have been shown to be quite marked as HIV patients with lower education and income levels have significantly poorer survival than their more advantaged counterparts [32].

Both race/ethnicity and socioeconomic deprivation contributed independently to disparities in HIV/AIDS mortality, and, within each deprivation level, blacks and Hispanics had higher mortality rates than whites. What might explain such disparities? One possible explanation is that, within each area-deprivation group, blacks and Hispanics remain worse off than whites as they have significantly lower education and income levels and higher rates of poverty, unemployment, and lack of health insurance [1, 27]. Secondly, in general, racial minorities in the USA have reduced access to medical care and are more likely to forego or delay needed medical care than whites [1, 27]. Racial composition as measured by the percent black population has been used as a proxy measure of racial segregation and discrimination in the USA and has been associated with racial disparities in access and use of healthcare services as well as with increased mortality rates and HIV-risk behaviors [33–35]. In addition to reflecting the possible discrimination/segregation effects, community racial composition might influence health and healthcare services through its impact on the supply of healthcare providers, lower access to public services or amenities, and severe socioeconomic disadvantage [33, 34]. Recent data from the American Community Survey show that in neighborhoods where the percentage of black population exceeded 50%, and rates of poverty, unemployment, and lack of car ownership were 2-3 times higher than those for the USA as a whole [24]. In our analysis, racial composition was associated with increased HIV/AIDS mortality rates for both the total and black populations, independent of socioeconomic deprivation, health insurance, and medical underservice. These findings suggest possible inequities in access to HIV-related services and treatment among minorities and those living in predominantly minority communities.

HIV/AIDS mortality has declined so impressively since the peak epidemic years of the mid-1990s that it was no longer a leading cause of death for the total USA population in 2011 [13]. It was the 8th leading cause of death in the USA in 2005 but does not currently figure among the 15 leading causes of death for the nation [1, 13, 36]. However, it remains as the 8th leading cause of death among those aged 25–44, the 10th leading cause for the total black population, and the 4th leading cause of death among black men aged 25–44 years [1, 11, 36]. In terms of years of potential life lost, a substantial disease burden with an estimated 1.1 million people living with HIV, an incidence of about 50,000 new HIV infections annually, and the presence of marked social disparities, HIV/AIDS remains a major threat to public health in the USA [1, 11, 17, 18].

Our long-term trend analyses of HIV/AIDS mortality in the USA according to race, SES, and geographical area represent an important contribution to the literature and build upon the previous US research on inequalities in HIV/AIDS mortality. However, this study has limitations. The deprivation indices used in our analysis were measured at the county level, which could vary greatly across census tracts or neighborhoods within a given county [3–6, 21, 27]. Unfortunately, census-tract geocodes are not available in the national mortality database, and the linkage of US mortality records and area deprivation is not feasible at the neighborhood level [3–6, 21, 27]. Given the compositional heterogeneity of counties, the effect of deprivation on HIV/AIDS mortality reported here is likely to be underestimated [3–6, 21, 27]. Secondly, our study was ecological in design, with county-level variations in mortality modeled as a function of a population-based ecologic variable, the area deprivation index. Although area-based socioeconomic differences in HIV/AIDS mortality are consistent with the expected pattern at the individual level, area-based patterns should not be considered as proxies for individual-level socioeconomic differentials. Such consideration may lead to the ecological fallacy [4–6, 21, 27].

A great deal of progress has been made to reduce HIV/AIDS mortality among all socioeconomic and demographic groups in the USA. However, despite the considerable mortality decline, the growing socioeconomic and racial disparities in HIV/AIDS mortality are a major public health concern; these disparities in mortality may partly indicate that the benefits of HAART have not been equitably distributed among all groups. The United States continues to have a 2-to-5-fold higher prevalence of HIV among adults than many OECD countries such as Canada, the United Kingdom, Australia, New Zealand, Germany, France, The Netherlands, Mexico, and Japan [37]. Health and social policy interventions such as improved access to health services, including more equitable access to HAART, and reductions in inequalities in education, poverty, unemployment, occupation, and housing are essential for tackling long-term health inequalities in HIV/AIDS mortality between socioeconomic and racial groups in the United States [3–5]. 

## Figures and Tables

**Table 1:** Trends in age-adjusted HIV/AIDS mortality rate per 100,000 population according to socioeconomic deprivation quintiles, United States, 1987–2009.

Year	Quintile 1		Quintiles 2–4		Quintile 5		Rate ratio and 95%		Rate ratio and 95%	
	(Low SES)		(Middle SES)		(High SES)		confidence interval		confidence interval	
	Deaths	Rate	Deaths	Rate	Deaths	Rate	Quintile 1/quintile 5		Quintiles (2–4)/quintile 5	
1987	3,529	7.86	7,895	5.53	2,041	4.06	1.94	1.83–2.04	1.36	1.30–1.43
1988	4,279	9.47	9,694	6.69	2,554	4.99	1.90	1.80–1.99	1.34	1.28–1.40
1989	5,338	11.81	13,249	9.02	3,388	6.42	1.84	1.76–1.92	1.40	1.35–1.46
1990	5,888	12.88	15,310	10.27	3,877	7.29	1.77	1.70–1.84	1.41	1.36–1.46
1991	6,991	15.11	17,840	11.75	4,605	8.50	1.78	1.71–1.84	1.38	1.34–1.43
1992	8,214	17.55	20,077	12.96	5,262	9.54	1.84	1.78–1.90	1.36	1.32–1.40
1993	9,060	19.10	22,370	14.22	5,827	10.37	1.84	1.78–1.90	1.37	1.33–1.41
1994	10,552	21.93	25,208	15.82	6,348	11.15	1.97	1.91–2.03	1.42	1.38–1.46
1995	10,900	22.27	25,582	15.82	6,625	11.50	1.94	1.88–2.00	1.38	1.34–1.41
1996	8,369	16.92	18,371	11.24	4,383	7.48	2.26	2.18–2.34	1.50	1.45–1.55
1997	4,803	9.63	9,602	5.83	2,105	3.56	2.71	2.57–2.84	1.64	1.56–1.71
1998	4,029	8.04	7,749	4.68	1,645	2.76	2.91	2.75–3.08	1.70	1.61–1.79
1999–2001	12,394	8.50	26,309	5.10	4,662	2.65	3.21	3.10–3.32	1.92	1.86–1.99
2002–2004	11,720	7.91	24,829	4.66	4,256	2.29	3.45	3.32–3.59	2.03	1.96–2.11
2005–2009	16,066	6.28	33,870	3.63	5,700	1.74	3.61	3.51–3.71	2.09	2.03–2.14

Source: Data derived from the US National Vital Statistics System, 1987–2009.

Death rates are age-adjusted by the direct method to the 2000 US standard population. The standard error (SE) of the age-adjusted mortality rate can be approximated by the rate divided by the number of deaths. For example, SE for the mortality rate in the most-deprived group in 2005–2009 = 6.28/SQRT (16,066) = 0.05. The standard normal *Z* test can be used to evaluate the statistical significance of the difference in mortality rates between any two groups at one point in time or to test for change in mortality rates between two time points for a specific group. If the absolute value of the Z-statistic = (R1 − R2)/SQRT [(SE(R1))^2^ + (SE(R2))^2^] ≥ 1.96, then the difference is statistically significant at the 0.05 level. If the test statistic ≥ 2.58, then the difference in the rates is significant at the 0.01 level, where R1 is mortality rate for the first demographic group, R2 is mortality rate for the second demographic group, and SE(R1) and SE(R2) are the standard errors associated with R1 and R2, respectively. For example, in 1995 the HIV/AIDS mortality rate in the most-deprived socioeconomic group (22.27) was 2.8 times higher than the corresponding rate in 1998 (8.04), and this difference was statistically significant at the 0.01 level (computed *Z*-value = 57.36).

The 95% confidence interval for a specific mortality rate can be constructed by using the formula = rate ± 1.96 ∗ SE.

**Table 2:** Weighted least squares regression models showing the impacts of the continuous socioeconomic deprivation index, urbanization level, primary medical care shortage areas, health uninsurance rate, and racial composition on county-level age-adjusted HIV/AIDS mortality rates, United States, 1992–1995 and 2003–2007 (*N* = 3141).

Covariate	Bivariate models					Multivariate models				
	*b*	**β**	*t*-stat.	*P* value	Adj. *R* ^2^	*b*	**β**	*t*-stat.	*P* value	Adj. *R* ^2^
Total US population, 2003–2007										
Socioeconomic deprivation index^1^	−0.08	−0.31	−18.47	<0.001	9.81	−0.04	−0.17	−8.14	<0.001	53.53
Urbanization level^2^	0.70	0.24	13.79	<0.001	5.69	0.62	0.21	13.9	<0.001	
Health professional shortage area^3^	2.81	0.20	11.24	<0.001	3.84	0.45	0.03	2.46	0.014	
Health uninsurance rate^4^	0.38	0.33	19.60	<0.001	10.91	0.10	0.08	4.72	<0.001	
Percent of black population	0.28	0.69	53.34	<0.001	47.53	0.24	0.58	42.83	<0.001	
Total black population, 2003–2007										
Socioeconomic deprivation index	−0.16	−0.21	−11.67	<0.001	4.20	−0.09	−0.11	−3.83	<0.001	17.07
Urbanization level	1.87	0.18	10.11	<0.001	3.16	2.67	0.26	13.44	<0.001	
Health professional shortage area	8.91	0.17	9.89	<0.001	3.02	3.14	0.06	3.54	<0.001	
Health uninsurance rate	0.98	0.23	13.42	<0.001	5.49	0.41	0.10	4.05	<0.001	
Percent of black population	0.29	0.31	17.83	<0.001	9.27	0.22	0.23	12.18	<0.001	
Black male, 2003–2007										
Socioeconomic deprivation index	−0.23	−0.23	−13.17	<0.001	5.32	−0.13	−0.13	−4.83	<0.001	24.50
Urbanization level	2.89	0.22	12.56	<0.001	4.83	4.03	0.31	16.86	<0.001	
Health professional shortage area	12.78	0.20	11.19	<0.001	3.87	4.36	0.07	4.07	<0.001	
Health uninsurance rate	1.38	0.26	15.00	<0.001	6.77	0.49	0.09	4.00	<0.001	
Percent of black population	0.46	0.38	22.54	<0.001	14.11	0.36	0.29	16.24	<0.001	
Black female, 2003–2007										
Socioeconomic deprivation index	−0.11	−0.18	−9.97	<0.001	3.13	−0.06	−0.09	−3.03	0.002	11.13
Urbanization level	1.19	0.14	7.83	<0.001	1.92	1.76	0.21	10.52	<0.001	
Health professional shortage area	5.86	0.14	8.06	<0.001	2.03	1.94	0.05	2.62	0.009	
Health uninsurance rate	0.69	0.21	11.66	<0.001	4.24	0.34	0.10	4.00	<0.001	
Percent of black population	0.18	0.24	13.50	<0.001	5.56	0.13	0.17	8.65	<0.001	
Total US population, 1992–1995										
Socioeconomic deprivation index	−0.11	−0.11	−6.41	<0.001	1.31	−0.07	−0.07	−2.95	0.003	30.85
Urbanization level	3.37	0.32	18.91	<0.001	10.35	3.18	0.30	15.39	<0.001	
Health professional shortage area	10.49	0.20	11.29	<0.001	3.94	3.23	0.06	3.87	<0.001	
Health uninsurance rate	1.26	0.30	17.47	<0.001	9.00	0.75	0.18	9.46	<0.001	
Percent of black population	0.64	0.44	27.35	<0.001	19.48	0.45	0.31	18.49	<0.001	
Total black population, 1992–1995										
Socioeconomic deprivation index	−0.01	−0.01	−0.18	0.854	0.00	−0.05	−0.03	−0.93	0.352	17.61
Urbanization level	8.66	0.34	19.34	<0.001	11.38	9.68	0.38	18.32	<0.001	
Health professional shortage area	23.26	0.17	9.08	<0.001	2.73	8.87	0.06	3.55	<0.001	
Health uninsurance rate	1.90	0.18	9.78	<0.001	3.19	2.02	0.19	8.52	<0.001	
Percent of black population	0.24	0.10	5.50	<0.001	1.00	0.13	0.05	2.72	<0.001	
Black male, 1992–1995										
Socioeconomic deprivation index	−0.03	−0.01	−0.48	0.631	0.01	−0.10	−0.03	−1.13	0.261	21.39
Urbanization level	14.94	0.37	21.27	<0.001	13.66	16.68	0.41	20.28	<0.001	
Health professional shortage area	40.67	0.18	10.01	<0.001	3.36	15.59	0.07	4.12	<0.001	
Health uninsurance rate	3.17	0.19	10.25	<0.001	3.56	3.07	0.18	8.36	<0.001	
Percent of black population	0.56	0.15	7.93	<0.001	2.12	0.37	0.10	4.99	<0.001	
Black female, 1992–1995										
Socioeconomic deprivation index	−0.03	−0.03	−1.46	0.144	0.08	−0.07	−0.06	−2.02	0.044	10.58
Urbanization level	3.62	0.25	13.45	<0.001	6.04	4.38	0.30	13.67	<0.001	
Health professional shortage area	9.25	0.12	6.14	<0.001	1.30	2.33	0.03	1.53	0.125	
Health uninsurance rate	1.02	0.17	8.96	<0.001	2.79	1.07	0.18	7.41	<0.001	
Percent of black population	0.23	0.10	5.22	<0.001	0.93	−0.02	−0.01	−0.56	0.574	

Notes: *b*: unstandardized regression coefficient; **β**: standardized regression coefficient; *R* ^2^: percentage variance explained.

*β* is also equal to the correlation coefficient in bivariate regression models.

^1^The 1990 or 2000 census socioeconomic deprivation index is a continuous variable with a mean of 100 and a standard deviation of 20. Higher index scores denote higher levels of socioeconomic position and lower levels of deprivation.

^2^The 2003 rural-urban continuum is used as a continuous variable, with code 1 being the most rural county and code 9 being the most urbanized county.

^3^A county is designated as a health professional shortage area for primary medical care services if it has a population to primary care physician ratio of at least 3,500 : 1; the reference groups are counties designated as nonshortage areas.

^4^The percentage of population without health insurance in 2000.

**Table 3:** Poisson regression models showing relative risk (RR) of HIV/AIDS mortality according to socioeconomic deprivation, urbanization level, primary medical care shortage areas, health uninsurance rate, and racial composition, United States, 1992–1995 and 2003–2007.

	2003–2007				1992–1995			
	Age-adjusted		Covariate-adjusted		Age-adjusted		Covariate-adjusted	
	RR	95% CI	RR	95% CI	RR	95% CI	RR	95% CI
Total US population								
Socioeconomic deprivation index								
Quintile 1 (low SES)	4.40	4.27–4.53	2.25	2.17–2.34	2.19	2.15–2.22	0.92	0.90–0.94
Quintiles 2–4 (middle SES)	2.13	2.07–2.19	1.68	1.64–1.73	1.44	1.42–1.46	0.90	0.89–0.92
Quintile 5 (high SES)	1.00	Reference	1.00	Reference	1.00	Reference	1.00	Reference
Urbanization level								
Large metro. counties^1^	3.20	2.87–3.57	3.88	3.48–4.33	4.04	3.71–4.40	6.73	6.18–7.33
Medium metro. counties^2^	2.18	1.95–2.43	2.98	2.67–3.33	2.43	2.23–2.65	4.93	4.53–5.38
Small metro counties^3^	1.58	1.41–1.77	2.09	1.86–2.34	1.68	1.53–1.83	3.31	3.04–3.62
Urban nonmetro. counties^4^	1.53	1.36–1.71	1.71	1.53–1.92	1.42	1.30–1.55	2.05	1.88–2.24
Rural counties^5^	1.00	Reference	1.00	Reference	1.00	Reference	1.00	Reference
Health professional shortage area								
Underserved	2.27	2.21–2.34	1.28	1.24–1.32	2.27	2.23–2.32	1.22	1.20–1.25
Not underserved	1.00	Reference	1.00	Reference	1.00	Reference	1.00	Reference
Health uninsurance rate^6^	1.44	1.43–1.45	1.14	1.12–1.15	1.39	1.39–1.40	1.45	1.44–1.46
Percent of black population^7^	1.50	1.49–1.51	1.38	1.38–1.39	1.33	1.32–1.33	1.28	1.27–1.28
Total black population								
Socioeconomic deprivation index								
Quintile 1 (low SES)	2.47	2.38–2.58	2.17	2.06–2.28	1.31	1.27–1.35	1.22	1.18–1.27
Quintiles 2–4 (middle SES)	1.63	1.57–1.69	1.55	1.49–1.61	1.02	1.00–1.05	0.95	0.93–0.98
Quintile 5 (high SES)	1.00	Reference	1.00	Reference	1.00	Reference	1.00	Reference
Urbanization level								
Large metro. counties	2.37	2.03–2.78	3.49	2.98–4.08	3.72	3.19–4.33	4.58	3.93–5.34
Medium metro. counties	1.94	1.66–2.28	2.95	2.51–3.46	2.49	2.13–2.90	3.36	2.88–3.92
Small metro. counties	1.46	1.24–1.71	2.05	1.74–2.41	1.52	1.30–1.78	1.95	1.67–2.29
Urban nonmetro. counties	1.54	1.31–1.81	1.73	1.47–2.03	1.44	1.23–1.69	1.56	1.34–1.83
Rural counties	1.00	Reference	1.00	Reference	1.00	Reference	1.00	Reference
Health professional shortage area								
Underserved	1.76	1.69–1.84	1.21	1.16–1.26	1.72	1.66–1.78	1.32	1.27–1.37
Not underserved	1.00	Reference	1.00	Reference	1.00	Reference	1.00	Reference
Health uninsurance rate	1.28	1.27–1.30	1.03	1.01–1.05	1.20	1.19–1.22	1.12	1.11–1.14
Percent of black population	1.17	1.16–1.18	1.10	1.10–1.11	1.08	1.07–1.08	1.03	1.02–1.04
Black male								
Socioeconomic deprivation index								
Quintile 1 (low SES)	2.42	2.30–2.54	2.10	1.97–2.24	1.32	1.28–1.36	1.23	1.18–1.29
Quintiles 2–4 (middle SES)	1.65	1.57–1.73	1.57	1.49–1.65	1.07	1.04–1.10	1.01	0.97–1.04
Quintile 5 (high SES)	1.00	Reference	1.00	Reference	1.00	Reference	1.00	Reference
Urbanization level								
Large metro. counties	2.48	2.04–3.00	3.59	2.96–4.35	3.85	3.23–4.59	4.71	3.95–5.62
Medium metro. counties	2.07	1.70–2.51	3.09	2.54–3.76	2.55	2.13–3.04	3.39	2.84–4.05
Small metro. counties	1.54	1.26–1.88	2.17	1.78–2.65	1.58	1.32–1.89	2.03	1.70–2.44
Urban nonmetro. counties	1.55	1.28–1.89	1.76	1.45–2.15	1.45	1.21–1.74	1.59	1.33–1.91
Rural counties	1.00	Reference	1.00	Reference	1.00	Reference	1.00	Reference
Health professional shortage area								
Underserved	1.74	1.65–1.83	1.19	1.13–1.26	1.75	1.68–1.83	1.32	1.27–1.38
Not underserved	1.00	Reference	1.00	Reference	1.00	Reference	1.00	Reference
Health uninsurance rate	1.27	1.25–1.29	1.02	1.01–1.05	1.19	1.17–1.20	1.10	1.08–1.11
Percent of black population	1.18	1.17–1.19	1.12	1.11–1.13	1.10	1.09–1.10	1.05	1.05–1.06
Black female								
Socioeconomic deprivation index								
Quintile 1 (low SES)	2.60	2.43–2.79	2.30	2.10–2.52	1.35	1.28–1.43	1.23	1.14–1.33
Quintiles 2–4 (middle SES)	1.61	1.51–1.72	1.53	1.42–1.65	0.93	0.88–0.98	0.84	0.79–0.89
Quintile 5 (high SES)	1.00	Reference	1.00	Reference	1.00	Reference	1.00	Reference
Urbanization level								
Large metro. counties	2.43	1.85–3.20	3.70	2.81–4.87	3.86	2.83–5.28	4.97	3.64–6.80
Medium metro. counties	1.85	1.41–2.44	2.91	2.20–3.84	2.48	1.81–3.39	3.61	2.63–4.94
Small metro. counties	1.36	1.02–1.81	1.94	1.46–2.58	1.38	1.00–1.91	1.82	1.32–2.53
Urban nonmetro. counties	1.53	1.16–2.03	1.71	1.29–2.27	1.40	1.01–1.92	1.48	1.08–2.05
Rural counties	1.00	Reference	1.00	Reference	1.00	Reference	1.00	Reference
Health professional shortage area								
Underserved	1.84	1.71–1.98	1.24	1.14–1.34	1.71	1.58–1.84	1.30	1.20–1.41
Not underserved	1.00	Reference	1.00	Reference	1.00	Reference	1.00	Reference
Health uninsurance rate	1.32	1.29–1.35	1.05	1.02–1.08	1.29	1.26–1.32	1.21	1.18–1.25
Percent of black population	1.16	1.15–1.18	1.08	1.07–1.10	1.06	1.05–1.07	0.99	0.98–1.00

^1^Counties in metropolitan areas with 1 million population or more. ^2^Counties in metropolitan areas of 250,000–1,000,000 population. ^3^Counties in metropolitan areas with population < 250,000. ^4^Urban nonmetropolitan counties consisting mostly of small urban towns with population < 20,000. ^5^Rural counties with no places with a population of ≥2,500. ^6^Relative risk associated with each 5-percentage-point increase in the proportion of population without health insurance. ^7^Relative risk associated with each 10-percentage-point increase in the proportion of the black population in a county.
